# No association between obstructive sleep apnea and restless legs syndrome: results from a large hospital-based cross-sectional study

**DOI:** 10.3389/frsle.2025.1632662

**Published:** 2025-10-21

**Authors:** Erik Vevatne Øverland, August Waaraas, Ragnhild S. Lundetræ, Sverre Lehmann, Ingvild W. Saxvig, Bjørn Bjorvatn

**Affiliations:** ^1^Department of Global Public Health and Primary Care, University of Bergen, Bergen, Norway; ^2^Centre for Sleep Medicine, Haukeland University Hospital, Bergen, Norway; ^3^Norwegian Competence Center for Sleep Disorders, Haukeland University Hospital, Bergen, Norway; ^4^Department of Clinical Science, University of Bergen, Bergen, Norway

**Keywords:** restless legs syndrome, obstructive sleep apnea, sleep disorders, prevalence, hospital-based study

## Abstract

**Introduction:**

Some studies have indicated a possible association between obstructive sleep apnea (OSA) and restless legs syndrome (RLS). Our aim was to explore this association in a large sample of patients referred to a hospital for suspected OSA.

**Methods:**

The sample included 8,852 patients referred to Haukeland University Hospital with suspicion of OSA between 2011 and 2022. OSA was diagnosed and categorized using standard respiratory polygraphy. Prior to the sleep study the patients completed an extensive questionnaire, including questions to determine if they had RLS. Pearson chi-square tests were used to examine RLS in relation to the presence and severity of OSA. Two separate logistic regression analyses were conducted. The first with moderate-severe OSA as the dependent variable and RLS as predictor, the second with RLS as the dependent variable and OSA severity as predictor. Both were adjusted for sex, age, marital status, alcohol consumption, daily smoking, caffeine after 17:00, and body mass index ≥30.

**Results:**

In total, 24.0% fulfilled the criteria for RLS, whereas moderate-severe OSA (apnea-hypopnea-index ≥15) occurred in 38.1% of the patients. The proportion of patients with RLS did not differ depending on OSA severity. Furthermore, there was no association between RLS and OSA in either chi-square or logistic regression analyses.

**Conclusion:**

The present study did not show increased prevalence of RLS in patients with OSA compared to patients without OSA. Furthermore, we found no increase in prevalence of RLS with increasing OSA severity. This suggests that these two sleep disorders are independent of each other.

## 1 Introduction

Obstructive sleep apnea (OSA) is a sleep disorder in which the patient experiences episodes of complete or partial collapse of the airways during sleep, usually with an associated decrease in oxygen levels or arousal from sleep (Lundetræ et al., 2018; Slowik et al., 2022). Symptoms typically include daytime sleepiness and snoring. OSA is associated with other diseases such as cardiovascular events, hypertension, diabetes mellitus type 2, mental illness, obesity, and reduced quality of life (Javaheri et al., 2017; Gleeson and McNicholas, 2022; Slowik et al., 2022). Furthermore, OSA is often comorbid with other sleep disorders, such as insomnia (Bjorvatn et al., 2015; Sweetman et al., 2022).

Restless legs syndrome (RLS) is a sleep disorder characterized by an unpleasant sensation or tingling that occurs at rest and is relieved by movement, such as walking (Gossard et al., 2021). Symptoms typically occur at night or late in the day and can thus contribute to difficulty initiating and maintaining sleep and may in some cases lead to excessive daytime sleepiness. This daytime sleepiness is, however, often described as less severe than the profound daytime sleepiness associated with other sleep conditions, such as narcolepsy. Lack of profound daytime sleepiness is thus listed as a supportive criterion for RLS according to the International Restless Legs Syndrome Study Group (IRLSSG) consensus criteria (Allen et al., 2014). Interestingly, whenever daytime sleepiness and fatigue are common, patients tend not to nap, implying hyperarousal in RLS (American Academy of Sleep Medicine, 2014). However, a recent study documented that RLS patients have both increased subjective and objective sleepiness compared to controls (Chenini et al., 2025). Similar to OSA, RLS is also often comorbid with insomnia (Vlasie et al., 2022).

Both OSA and RLS are known to be among the most common sleep disorders. Regarding OSA, the prevalence varies between studies, with rates ranging from 10 to 50% in males, and 9–23% in females, depending on severity criteria and other methodological differences among studies. It is estimated that 425 million people globally suffer from moderate to severe OSA (Rundo, 2019; Slowik et al., 2022). A number of studies estimate that RLS affects up to 10% of the general population and is severe in 2–3% (Bjorvatn et al., 2005; Romero-Peralta et al., 2019; Gossard et al., 2021). Both diagnoses are believed to be underdiagnosed and undertreated (Romero-Peralta et al., 2019; Rundo, 2019). Interestingly, OSA is more common in men, whilst RLS is more common in women (Ohayon and Roth, 2002; Slowik et al., 2022).

Surprisingly, not many studies have investigated whether there is a relationship between OSA and RLS. Both OSA and RLS are known to be associated with increased systemic inflammation (Kheirandish-Gozal and Gozal, 2019; Jiménez-Jiménez et al., 2023). This heightened inflammatory signaling may possibly suggest that the two sleep disorders may be associated with each other. Another possible link between the two conditions is hypoxia. One study reported peripheral hypoxia to be positively correlated with symptoms of RLS, and it would be plausible that hypoxia related to OSA could cause symptoms of RLS (Salminen et al., 2014). This is collaborated in a small prospective study from 2005 which showed that the prevalence of clinically significant RLS in patients with clinically significant OSA was 8.3%, compared to 2.5% in a comparable control group (Lakshminarayanan et al., 2005). Another study based on phone interviews also concluded that OSA was associated with RLS (Ohayon and Roth, 2002). However, the latter study did not have an objective assessment of OSA. A third study from 2015 postulates that OSA is related to RLS during pregnancy (Terzi et al., 2015). This study, however, included only a small number of participants, and was based on self-reported symptoms of OSA in face-to-face interviews. On the other hand, a recent small study involving 326 people showed no significant association between OSA and RLS (Gothi et al., 2023). Accordingly, there is need for more studies that investigate the association between OSA and RLS with standardized and objective registrations of OSA, and in large samples of patients.

In light of this context, the current study had two main objectives: Firstly, to evaluate the prevalence of RLS among a large group of patients referred to a university hospital with suspected OSA. Secondly, to assess the prevalence of RLS in relation to the severity of OSA within this cohort of patients. Our main hypothesis, based on available literature, was that RLS would be associated with OSA. We also hypothesized that the severity of OSA was positively associated with RLS.

## 2 Materials and methods

The sample included 8,852 patients referred to the Centre for Sleep Medicine at Haukeland University Hospital between 2011 and 2022. All the patients included in the sample were referred due to suspicion of OSA. The patients included in the study all had valid data from sleep recordings. Only patients above 16 years who consented to participate were included.

The patients were investigated by a standard respiratory polygraphic sleep study using a type 3 portable monitor (Embletta™ or NOX T3, Resmed Norway AS). Most of the patients were recorded as they slept at home and the rest slept in a hospital hotel. The scoring guidelines followed the 2007 American Academy of Sleep Medicine manual (Conrad Iber, 2007), which defines apneas as a reduction of 90% or more of baseline nasal airflow with a duration of at least 10 s. Hypopneas were defined as a nasal airflow reduction of 30–90% relative to baseline, lasting at least 10 s coupled with a decrease in oxygen saturation of ≥4%. The diagnosis of OSA was classified in accordance with the apnea-hypopnea index (AHI): No OSA (< 5); mild OSA (5–14.9); moderate OSA (15–29.9); severe OSA (≥30).

Before the respiratory sleep study, as part of the diagnostic workup, the patients completed an elaborate questionnaire that covered a wide range of topics in sleep medicine. During the consultation, a nurse or doctor reviewed the questionnaire to ensure the reliability of the answers, making adjustments if needed. This study focused on the subset of questions in the questionnaire related to RLS, based on the validated Hopkins telephone diagnostic interview (Hening et al., 2009). The questions asked were:

1. Do you have an urge to move your legs or arms, normally related to a discomfort or indeterminate tingling in your legs or arms?
2. If yes, does this urge start or increase when you are at rest, for example when lying down or sitting still?
3. Is the urge to move or the uncomfortable tingling partially or completely gone when you are moving, for example when you are walking?
4. Is the urge to move or the uncomfortable tingling worse late in the day or in the night than the rest of the day?

The questions had three response alternatives (no, yes, and don't know). In the statistical analyses the RLS variables were dichotomized into RLS (yes to all four questions) and not RLS (“no” or “don't know” to any of the four questions). To be classified as having RLS, the patients had to answer “yes” to all four RLS questions. If one or more of the questions were answered with “no” or “don't know” or left unanswered, the patients were classified as not having RLS. There were some missing data regarding the RLS questions, but we excluded only patients who did not answer any of the four RLS questions.

Answers regarding sex, age, marital status/cohabitation, alcohol consumption, smoking, caffeine consumption after 17:00 and body height and weight (measured by nurses at the clinic) were part of the analysis. Marital status was dichotomized into living alone or cohabitating/married. Alcohol was categorized into 3 groups (never/rarely consume alcohol, 1–2 times per week and ≥3 times per week). Caffeine consumption after 17:00 was dichotomized into 0 or ≥1 cups of caffeine containing beverages. Smoking was dichotomized into 0 cigarettes per day or ≥1 cigarette per day. Lastly, body mass index (BMI) was calculated using the formula: weight (kg)/[height (m)]^2^ and further dichotomized into BMI < 30 or ≥30.

### 2.1 Ethics

The research received approval from the Regional Committee for Medical and Health Research Ethics, Health Region West (REK 2014/1060). All patients provided written informed consent prior to their participation in the study.

### 2.2. Statistical analysis

The data analyses were conducted using IBM SPSS Statistics, version 28.0. Pearson chi-square tests were employed to examine variances in patient characteristics categorized by the presence and severity of OSA. Logistic regression analyses were conducted with moderate-to-severe OSA as dependent variable and sex, age, alcohol consumption, marital status, BMI, caffeine consumption after 17:00, daily smoking, and RLS as predictors. Separate logistic regression analyses were conducted with RLS as the dependent variable and mild-, moderate- and severe OSA as predictors. Both models were conducted crude as well as adjusted for age, sex, marital status, alcohol consumption, daily smoking, caffeine after 17:00, and BMI ≥30. The adjusting variables were chosen due to their established associations with OSA, RLS, or both (Ohayon and Roth, 2002; Fuhrman et al., 2012; Rundo, 2019; Antonaglia and Passuti, 2022; Conde et al., 2022; Pataka et al., 2022; Kawada, 2023; Patial et al., 2023; Ko et al., 2024). Significance level was set to 0.05 for all analyses.

## 3 Results

Among the 8,852 patients referred to the hospital with suspected OSA and a valid polygraphic recording, 2,847 patients were females and 6005 were males. The mean age was 49.5 ± SD 14.0 (range 16–89) years, and 3,834 (44.5%) of the study population had obesity (BMI ≥30). A total of 972 patients were excluded from the RLS analyses due to no responses to the four RLS-related questions. RLS was present in 24.0%. Compared to the analytic sample, the excluded patients were older (chi-square, *p* < 0.001), more often females (*p* = 0.011), and had more often moderate-severe OSA (*p* < 0.001), but marital status (*p* = 0.51) was not significantly different.

Moderate-severe OSA (AHI ≥15) occurred in 3,372 (38.1%) patients (Table 1).

**Table 1:** Background characteristics of 8,852 patients referred with suspected obstructive sleep apnea (OSA) to Haukeland University Hospital in Bergen, Norway.

**Characteristic**	***n* (%)**	**Missing, *n* (%)**
**Sex:**		
Male	6,005 (67.8)	0 (0.0)
Female	2,847 (32.2)	0 (0.0)
**Age in years:**		
< 30	972 (11.0)	0 (0.0)
30–49	3,317 (37.5)	
50–69	3,912 (44.2)	
>70	651 (7.4)	
**Married/cohabiting:**		
No	2,027 (25.5)	897 (10.1)
Yes	5,928 (74.5)	
**Alcohol consumption:**		
Never/rarely	4,939 (61.8)	861 (9.7)
1–2 days per week	2,473 (30.9)	
3 or more days per week	579 (7.2)	
**Daily smoking:**		
No	6,170 (76.9)	831 (9.4)
Yes	1,851 (23.1)	
**Caffeine after 17:00:**		
No	3,117 (38.8)	809 (9.1)
Yes	4,926 (61.2)	
**BMI** >**30:**		
No	4,791 (55.5)	227 (2.6)
Yes	3,834 (44.5)	
**OSA severity:**		
No OSA (AHI < 5)	2,496 (28.2)	0 (0.0)
Mild OSA (AHI 5–14.99)	2,984 (33.7)	
Moderate OSA (AHI 15–29.9)	1,821 (20.6)	
Severe OSA (AHI ≥ 30)	1,551 (17.5)	
**RLS:**		
No	5,992 (76.0)	972 (11.0)
Yes	1,888 (24.0)	

AHI, Apnea-hypopnea index; OSA, obstructive sleep apnea.

We found no correlation between RLS and OSA (Table 2). The proportion of patients with RLS did not increase with higher OSA severity. However, there was an association between RLS and sex, with more females than males reporting RLS. Furthermore, increasing age was associated with a higher prevalence of RLS (Table 2). Patients with obesity (BMI ≥30) had a higher prevalence of RLS compared to non-obese patients. Being married/cohabitating and smoking were also positively associated with RLS. On the other hand, alcohol and caffeine consumption showed no association with RLS (Table 2).

**Table 2:** Association between restless legs syndrome and various factors including severity levels of obstructive sleep apnea, sex, age, marital status, alcohol consumption, daily smoking, caffeine after 17:00, and BMI ≥30.among patients referred with suspected sleep apnea to Haukeland University Hospital in Bergen, Norway (*n* = 8,088).

**Variable**	**RLS, *n* (%)**	**Chi-square (df)**	***p*-value**
**OSA severity:**			
No OSA (AHI < 5)	529 (23.0)	3.91 (3)	0.27
Mild OSA (AHI 5–14.99)	652 (24.4)		
Moderate OSA (AHI 15–29.9)	399 (25.4)		
Severe OSA (AHI > 30)	308 (23.1)		
**Sex**			
Female	730 (28.5)	39.80 (1)	**<** **0.001**
Male	1,216 (22.0)		
**Age**			
< 30	146 (16.2)	50.53 (3)	**<** **0.001**
30–49	706 (23.0)		
50–69	966 (27.1)		
>70	128 (23.3)		
**Marital status**			
Married/cohabiting	1,455 (24.7)	5.93 (1)	**0.015**
Single	449 (22.0)		
**Alcohol consumption:**			
Never/rarely	1,178 (23.9)	1.35 (2)	0.51
1–2 days per week	614 (24.8)		
3 or more days per week	134 (22.9)		
**Daily smoking:**			
No	1,416 (22.9)	20.57 (1)	**<** **0.001**
Yes	512 (28.1)		
**Caffeine consumption:**			
Caffeine after 17:00	1,196 (24.5)	0.78 (1)	0.38
No caffeine after 17:00	740 (23.6)		
**BMI**			
< 30	1,014 (22.9)		
≥30	878 (25.5)	7.33 (1)	**0.007**

Patients referred to the study who failed to respond to any of the questions pertaining to Restless Legs Syndrome (RLS) were excluded from the analysis. DF, degrees of freedom; OSA, obstructive sleep apnea; AHI, apnea-hypopnea index; BMI, Body mass index. Significant findings are indicated in bold text.

Adjusted logistic regressions indicated that male sex, older age, living alone, caffeine after 17:00, and obesity were all significant predictors for moderate-severe OSA. There was no association between RLS and moderate-severe OSA in neither crude nor adjusted analyses (Table 3).

**Table 3:** Logistic regression analysis with moderate-to-severe obstructive sleep apnea (OSA) as dependent variable among 8,852 patients referred to Haukeland University Hospital in Norway with suspected OSA.

**Category (*n*)**	**Moderate-severe OSA**	
	**Crude OR (95% CI)**	**Adjusted**^a^ **OR (95% CI)**
**Sex:**		
Female (2,847)	1.00	1.00
Male (6,005)	**1.95 (1.77–2.15)**	**2.49 (2.21–2.80)**
**Age:**		
< 30 years (972)	1.00	1.00
30–49 years (3,317)	**3.24 (2.62–4.00)**	**3.34 (2.62–4.27)**
50–69 years (3,912)	**7.49 (6.08–9.23)**	**9.13 (7.16–11.64)**
>70 years (651)	**10.99 (8.54–14.14)**	**14.64 (10.85–19.75)**
**Marital status:**		
Married/cohabiting (5,928)	1.00	1.00
Single (2,027)	0.94 (0.84–1.04)	**1.32 (1.17–1.50)**
**Alcohol consumption:**		
Never/rarely (4,939)	1.00	1.00
1–2 days per week (2,473)	1.09 (0.99–1.20)	1.02 (0.91–1.15)
3 or more days per week (579)	**1.54 (1.30–1.83)**	1.10 (0.91–1.34)
**Daily smoking:**		
No (6,170)	1.00	1.00
Yes (1,851)	**0.89 (0.80–0.997)**	0.99 (0.87–1.13)
**Caffeine after 17:00 h:**		
No (3,117)	1.00	1.00
Yes (4,926)	**1.23 (1.12–1.35)**	**1.13 (1.02–1.26)**
**BMI:**		
< 30 (4,791)	1.00	1.00
≥30 (3,834)	**2.23 (2.04–2.44)**	**2.61 (2.35–2.90)**
**Restless legs syndrome:**		
No (5,992)	1.00	
Yes (1,888)	1.03 (0.93–1.15)	1.00 (0.88–1.12)

Referred patients with missing objective data regarding OSA were excluded from this analysis. OSA was dichotomized into no OSA and mild OSA as one category and moderate OSA and severe OSA as the other category. OSA, Obstructive sleep apnea, OR, odds ratio; CI, confidence Interval; BMI, Body Mass Index. Significant findings are indicated in bold text.

^a^Adjusted for sex, age, marital status, alcohol consumption, daily smoking, caffeine after 17:00, and BMI ≥ 30.

Furthermore, when RLS was the dependent variable with OSA severity as predictor, we found no significant associations. This was true for both crude and adjusted analyses (Table 4).

**Table 4:** Logistic regression with restless legs syndrome as the dependent variable and obstructive sleep apnea severity as predictor among 8,088 patients referred to Haukeland University Hospital in Norway.

**OSA severity**	**Restless legs syndrome**	
	**Crude OR (95% CI)**	**Adjusted OR**^a^ **(95% CI)**
Mild OSA	1.00	1.00
Moderate OSA	1.05 (0.91–1.22)	1.05 (0.90–1.23)
Severe OSA	0.93 (0.80–1.08)	0.94 (0.80–1.12)

Referred patients that did not answer any of the questions about RLS were excluded from this analysis (*n* = 972). OR, odds ratio; CI, confidence interval; OSA, obstructive sleep apnea.

^a^Adjusted for sex, age, marital status, alcohol consumption, daily smoking, caffeine after 17:00, and BMI ≥ 30.

## 4 Discussion

In this large-scale study on patients referred to a hospital with suspicion of OSA, we found no significant increase in the prevalence of RLS in the patients with OSA compared to those not having OSA. This was in contrast with our initial hypothesis of RLS being more prevalent among patients with OSA compared to those without OSA. Our second hypothesis, that increasing OSA severity would be a predictor for RLS was also not confirmed. In fact, the prevalence of RLS decreased as OSA severity increased from moderate to severe, compared to mild to moderate OSA. Although these findings were not statistically significant, the absence of a clear trend further disproves our hypothesis.

Some available literature postulates that RLS is common in patients with OSA (Ohayon and Roth, 2002; Lakshminarayanan et al., 2005; Terzi et al., 2015). In the current study it was therefore surprising to find no association between RLS and OSA. We found a strong association between OSA and male sex. This is consistent with already available literature (Bonsignore et al., 2019; Rundo, 2019; Antonaglia and Passuti, 2022). The reason for the strong association between OSA and male sex may be due to differences in obesity, upper airway anatomy, breathing control, hormones or aging. In the present study, we adjusted for BMI and age when studying the association between OSA and RLS.

The strong association between RLS and female sex was an expected finding based on the available literature (Ohayon and Roth, 2002; Kawada, 2023). The reason for this is not fully understood and probably multifactorial. Possible explanations are pregnancy, hormonal factors and lower iron in serum (Theorell-Haglöw et al., 2018). Another explanation could be that RLS causes more sleepiness in women and thus prompts an investigation for OSA where RLS is found incidentally (Holzknecht et al., 2019). Furthermore, OSA was strongly associated with increasing age. This was also as expected (Rundo, 2019; Lee and Sundar, 2021). The reason for this is also believed to be multifactorial. Some hypotheses are that aging leads to reduced response to hypoxia as well as reduced lumen of the upper airway, resulting in higher prevalence of sleep apnea (Glasser et al., 2011). We also found RLS to be more prevalent in obese patients. This is consistent with findings from other studies as reported in a meta-analysis by Lin et al. studying the association between obesity and RLS. Several mechanisms are suggested for explaining this link, such as increased risk of poor vascular endothelial function, increased risk of brain- and systemic iron deficiency leading to hypoxia and myelin loss, and fewer dopamine D2 receptors compared with controls, which are all seen at higher prevalences in obese patients (Lin et al., 2018).

Several studies have aimed to find the prevalence of RLS in the general population (Ohayon et al., 2012). Ohayon et al. conducted a review of 47 studies investigating the prevalence of RLS and found a prevalence between 3.9% and 14.3% when using the same diagnostic criteria as used in our study. In contrast, in our study population, we found that 24.0% had RLS. It is crucial to note that the patient group in our study cannot be compared to the general population. All patients included in this study were referred to the hospital due to suspected OSA, indicating that they all presented with symptoms or complaints of poor sleep such as snoring, breathing pauses during sleep, fatigue, and not least daytime sleepiness. This may explain why we find a higher prevalence of RLS in our study population, as these patients are already under suspicion of having pathological sleep. A study by Gothi et al. on a similar patient population with patients referred for polygraphy or polysomnography showed a prevalence of RLS of 24.4% in the OSA group and 28.5% in the non-OSA group (Gothi et al., 2023), in line with our findings. This suggests that the high prevalence of RLS in this group of patients is not unprecedented. Another possible explanation to the high prevalence of RLS could be that our diagnosis was based on self-reporting rather than interviews. A study conducted amongst patients visiting their general practitioner found the prevalence of RLS to be 14.3%, using a similar method of diagnosing as used in the present study, potentially indicating higher prevalences from self-reports than interviews (Bjorvatn et al., 2021). Furthermore, we did not gather information on other conditions that could mimic RLS, such as peripheral neuropathy or akathisia, which may have led to overdiagnosing RLS.

OSA and RLS are associated with increased systemic inflammation (Kheirandish-Gozal and Gozal, 2019; Jiménez-Jiménez et al., 2023). This heightened inflammatory signaling suggests a possible association between the two sleep disorders. In line with this, some studies show an association between OSA and RLS (Ohayon and Roth, 2002; Lakshminarayanan et al., 2005; Terzi et al., 2015). Another possible shared mechanism between OSA and RLS is hypoxia. A study investigating the association between peripheral hypoxia and RLS symptom severity found a positive correlation, indicating that hypoxia may be part of the pathophysiological mechanism of RLS (Salminen et al., 2014). One could speculate that the global hypoxia associated with OSA could trigger symptoms of RLS. However, as the hypoxia associated with OSA occurs while the patient is asleep, the increase in subjective RLS symptoms may not be noticed by the patient and thus not reported by the patients in our study.

Another study done with 326 people showed no association between OSA and RLS (Gothi et al., 2023), similar to our findings. A potential reason for the lack of association between OSA and RLS might be their other differences in underlying pathophysiological mechanisms. OSA is characterized by recurrent collapse of the pharyngeal airways during sleep due to anatomical features like narrow upper airways (Haponik et al., 1983; Burger et al., 1992; Schwab et al., 1995), adipose deposits in the pharynx (Horner et al., 1989; Shelton et al., 1993) and the arrangement of the soft tissue surrounding the pharynx (Schwab et al., 1995). In contrast, although the exact pathophysiological mechanism of RLS is not fully understood, RLS seems to rely on dopaminergic dysfunction and changes in CNS iron homeostasis (Trenkwalder et al., 2009). The lack of overlap between the underlying mechanisms for the two disorders can explain why there was no association between the two conditions.

The adjusted analyses indicated that living alone increased the risk of having moderate-severe OSA. Other studies have found that in the general population, cohabitation is a risk factor for OSA (Fuhrman et al., 2012). We postulate that the reason for this apparent discrepancy is that our study population was already referred with suspicion of OSA and a person without a bedpartner will on average have a higher burden of symptoms when they decide to see their doctor. Thus, they will be more likely to have a more severe degree of OSA. The adjusted analyses indicated that alcohol consumption did not increase the risk of moderate-severe OSA. This is contrary to findings from other studies that show that increased alcohol intake may lead to an increased risk of OSA (Ko et al., 2024). The reason for this discrepancy is unclear.

It is important to acknowledge both the strengths and the limitations of this study. The sample in the present study was large, enabling adjustment for relevant confounding factors. Moreover, more than 90% of the patients referred to the hospital with suspected OSA agreed to participate, leading to a representative sample of those referred for OSA assessment. Another significant strength of this study was the use of objective AHI measures, which eliminated the influence of common method bias when comparing these data with patient self-reports (Podsakoff et al., 2003). One limitation of this study was the use of polygraphy rather than polysomnography for diagnosing OSA. This could be problematic, as AHI tends to be underestimated in polygraphic recordings compared to polysomnography (Masa et al., 2011; Nerfeldt et al., 2014). The assessment of RLS was based on self-reports, but all patients had a doctor or nurse available if any questions in the questionnaire was unclear. Our study did not include an independent control group from the general population. This would have strengthened our study as the patients in our control group all had some sleep-related symptoms, making them distinct from the general population. We did not explore the potential shared mechanism of hypoxia in our study or analyze RLS symptoms based on measures such as oxygen desaturation index or mean saO_2_ levels. This could potentially be an intriguing topic for further research. The patients excluded from analysis were older, more likely to be female and had more severe OSA. This should be kept in mind when interpreting the data. Another limitation was that other possible confounders such as diabetes, iron deficiency, and medication use were not considered. Furthermore, one limitation was that we did not collect information about RLS mimics such as peripheral neuropathy and akathisia. This may have impacted the reported prevalence of RLS.

The present study population was referred to the hospital with suspicion of OSA. We recommend that future investigations should be conducted in more diverse populations to confirm the findings. Regarding implications for clinical practice, this study shows that there seems to be no need for RLS screening solely based on the presence of OSA and vice-versa.

### 4.1 Conclusion

The present large, hospital-based, cross-sectional study sought to determine whether an association exists between OSA and RLS in a population of patients referred for suspected OSA. Contrary to some previous studies, we did not find any significant association between RLS and OSA, nor did the severity of OSA appear to predict the presence of RLS. These results suggest that RLS and OSA might have distinct underlying pathophysiological mechanisms. Considering the study's limitation of lacking a control group reflective of the general population, future research should aim to confirm these findings in a broader, more diverse population. Clinically, these findings indicate that RLS screening in patients with OSA may not be necessary unless other risk factors are present.

## Data Availability

The datasets presented in this article are not readily available but the data underlying this article will be shared at reasonable request to the corresponding author. Requests to access the datasets should be directed to Erik Vevatne Øverland, red008@uib.no.
